# Ultrasound anatomy of the transversus abdominis plane region in pregnant women before and after cesarean delivery

**DOI:** 10.1186/s12871-016-0289-8

**Published:** 2016-12-22

**Authors:** Nicholas Kiefer, Stefanie Krahe, Ulrich Gembruch, Stefan Weber

**Affiliations:** 1 Department of Anesthesiology and Intensive Care Medicine, University of Bonn Medical Center, Bonn, Germany; 2 Department of Obstetrics and Prenatal Medicine, University of Bonn Medical Center, Bonn, Germany

**Keywords:** Ultrasound, Cesarean delivery, Transversus abdominis plane block

## Abstract

**Background:**

After cesarean delivery, analgesia is often incomplete and a multimodal approach to analgesia is necessary. Transverse abdominal plane (TAP) block has been advocated in this setting, yet no systematic description of the ultrasound anatomy in pregnant women exists in the literature. Therefore, we aimed to describe the sonographical features of relevant structures in pregnant women before and after elective cesarean.

**Methods:**

Sixty women at, or close to term scheduled for elective cesarean delivery underwent a standardized ultrasound examination before and after delivery. We assessed the visibility of the muscular layers and measured the distance from the skin to the layers of the abdominal wall muscles in the region for TAP block before and after cesarean section on both side.

**Results:**

The three muscular layers of the lateral abdominal wall (external oblique, internal oblique and transversus abdominis muscle) were visible in all examinations. Before cesarean section the median TAP distance was shorter: 2.9 cm (interquartile range 2.6–3.6) compared to 3.9 cm (3.1–4.5) after cesarean section (left side, *p* < 0.001). The external and internal oblique muscles were located closer to the skin surface before cesarean section. An increased body mass is associated with increased the TAP distance before and after birth (*p* < 0.001).

**Conclusion:**

Relevant anatomical landmarks for a TAP block are sonographically well visible after cesarean delivery. Postoperatively, depth of the TAP as compared to before birth is increased significantly. Scanning the abdominal wall before CD will underestimate the target depth of the TAP after delivery. The obstetric anesthetist needs to be aware of these changes when planning a TAP block in the context of cesarean delivery.

## Background

Worldwide, the rate of cesarean deliveries (CD) has greatly increased in the last decades and keeps rising [1–3]. After CD analgesia is often incomplete with women reporting unacceptably high average pain scores. At 24 h a median visual analog scale for pain has been reported and 85% of women are reporting that the pain interferes with one or more daily activities [4, 5]. Severe pain after childbirth may be associated with chronic pain and post-partum depression [5–7]. Thus, inadequate analgesia after CD is a very frequent and medically important issue and may involve severe consequences in the individual patient. This has generated interest in enhanced analgesic concepts. Undisputedly, intrathecal opioids are the mainstay of pain relief if cesarean section is performed under spinal anesthesia, but the duration of a single shot spinal anesthesia is limited, wherefore a multimodal approach is necessary. This may include a combined spinal-epidural anesthesia, postoperative opioids or a transverse abdominal plane (TAP) block. The TAP block has been demonstrated to be effective in reducing post-operative pain scores and side effects of opioids in individual trials after CD [8, 9]. The original technique was described by Rafi and uses a landmark approach [10]. Although the landmark approach is known as to be effective, safety issues have been raised and an ultrasound guided approach has been advocated instead [11]. No systematic data on the ultrasound anatomy of the abdominal wall in pregnant women at term have been published so far.

Usually, TAP blocks are initiated after cesarean delivery [12]. We hypothesized that childbirth affects the sonographical features of the abdominal wall. Therefore, we prospectively assessed visibility and distance between skin and TAP (TAP distance) on both sides before and after elective CD in pregnant women undergoing elective CD at, or close to term (≥35 Weeks of gestation).

## Methods

Pregnant women at, or close to term (≥ 35 weeks of gestation) scheduled for elective CD under spinal anesthesia were enrolled into this prospective, observational study. Ethics approval and consent are described in detail in the declarations section at the end of the manuscript. The women underwent two standardized ultrasound examinations, all performed by a senior anesthetist, experienced in obstetrical anesthesia as well as TAP blocks, using a LOGIQe ultrasound machine (GE Healthcare, Chalfont St Giles, UK) with the 12 l-RS linear array probe (5–13 MHz). The first ultrasound examination was scheduled after the induction of spinal anesthesia immediately before the beginning of surgical preparation. The patient was positioned in a lithotomy position with her legs in leg holders (Goepel knee crutch, Maquet, Rastatt, Germany) and the knees approximately at the level of the abdominal wall and with 10° left lateral displacement to avoid a supine hypotensive syndrome. The second examination was carried out in the same moderate lithotomy position of the patient but without lateral displacement after the end of all surgical procedures before moving the patient from the operating table to her bed. For each examination, the factory preset for soft tissue and identical settings for depth and focus were used. Loops and images derived from a standardized sequence were recorded and analyzed offline with the tools provided in the ultrasound machine. For comparability, the smallest possible pressure was applied to the transducer.

**Fig. 1:**
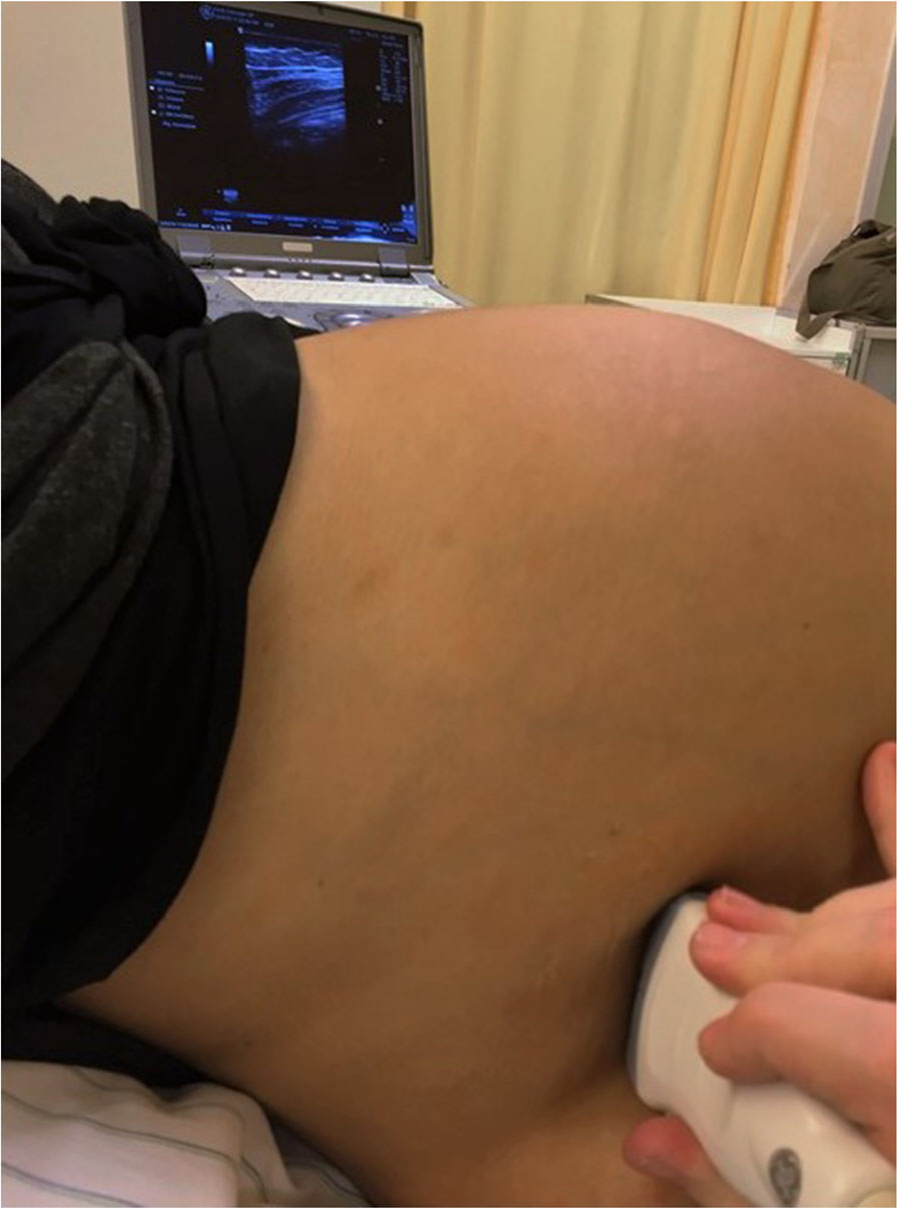
Position of the linear transducer to evaluate the muscular layers for a lateral TAP block in a pregnant woman at term

**Fig. 2:**
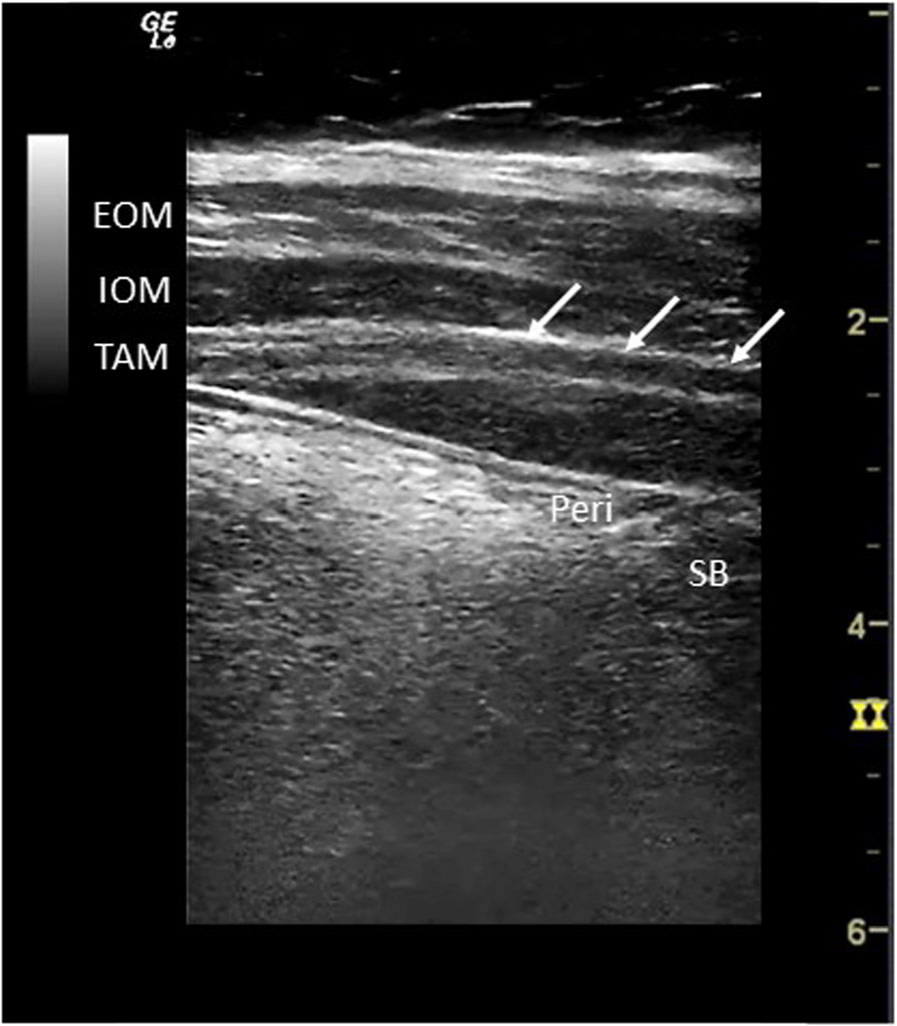
Typical sonogram of the abdominal wall in the lateral TAP position. Arrows indicate the TAP. EOM = external oblique muscle, IOM = internal oblique muscle, TAM transverse abdominal muscle, Peri = peritoneum, SB = small bowel

The transducer was placed above the iliac crest and below the rib cage between the anterior and middle axillary line as used for postoperative analgesia after CD [13]. A representative situation is shown in Fig. 1. According to current nomenclature this position corresponds to a lateral TAP position [14]. The orientation of the transducer was in the transverse plane and perpendicular to the skin. Both left and right side were assessed. The external oblique muscle is the muscle of the abdominal wall located closest to the skin, followed by the internal oblique and the transversus abdominis muscle. The transversus abdominis plane (TAP) is the outer border of the transversus abdominis muscle (Fig. 2). We assessed in how many cases all three muscular layers of the abdominal were visible in one single still frame (visibility). The perpendicular distance between skin and the external oblique muscle, the internal oblique muscle and the transversus abdominis plane (TAP) were measured in the center of the still frame image. All data was entered manually into a SPSS data sheet (SPSS 22, IBM North Caste, NY, USA). Demographic data that typically follows a normal distribution are displayed as mean ± standard deviation without test for normal distribution. Weeks of gestation and number of gravidity and parity are given as median and interquartile range. Demographic data were tested for significant differences versus data obtained from a database containing all parturients undergoing CD between 2009 and 2011 in the same institution with the non-parametric Mann-Whitney *U* Test because of largely different sample size. The number of gravidity and parity was tested for significant differences with Fishers exact test because of the low frequency in high and low values.

The TAP distance data were tested with the Kolomogorov-Smirnov Test for normal distribution after pooling data from both sides to avoid multiple testing. In order to guarantee a homogenous reporting style, all data are reported as median (interquartile range). Distances to muscular layers on the left and the right side before and after CD were compared using the Wilcoxon test for paired data. The SPSS UNIANOVA tool was used to identify factors that significantly affect TAP distance. Influence of body mass index (BMI) and the parity on TAP distance were then individually tested using a mixed linear model for paired data. For this analysis data from both and right side were pooled. For all statistical tests, a *p* < 0.05 was considered to be statistically significant. The box and whiskers of the box plot followed the definitions of Tukey [15]. The line within the box represents the median, the upper and lower limits of the box represent the first and third quartile, and thus, the length of the box represents the interquartile range (IQR). The whiskers represent the lowest value still within 1.5 × IQR of the lower quartile, and the highest value still within 1.5 × IQR of the upper quartile.

## Results

Between June 2012 and March 2013, 60 patients were enrolled into this study. Their demographic data and the data of 1362 patients undergoing elective CD in the same institution between 2009 and 2011 are provided in Table 1. Except for weeks of gestation at delivery, the study population did not differ significantly from the entirety of women delivering through a CD at term in this institution.

**Table 1:** Demographic data

	Study population	All CD	*p*
n	60	1362	
age (years)	34 ± 5	33 ± 6	0.56
weeks of gestation	39 (2)	38 (1)	< 0.001
gravidity/parity	2 (2)/2 (1)	2 (2)/2 (1)	0.98/0.07
height (cm)	168 ± 6	167 ± 7	0.35
baseline BMI	25 ± 6	25 ± 5	0.33
weight gain (kg)	13 ± 6	14 ± 6	0.14

Data presented as absolute numbers or mean ± standard deviation except gravidity/parity, which is presented as median (interquartile range), *BMI* body mass index

**Fig. 3:**
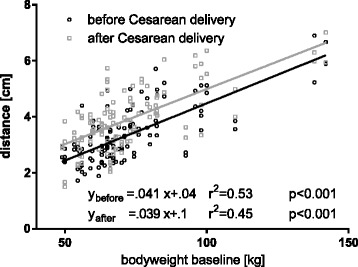
Distance from skin to TAP vs. body weight prior to pregnancy before (○) and after (□) CD. Data from left and right side are pooled

The relevant structures were visible in 100% of all exams in one single frame before, as well as after CD. The perpendicular distances the skin to the outer border of the relevant structures are displayed in Table 2. The median difference in distance of the TAP after versus before CD was 0.65 (0.1–2.2) cm. Before CD the right TAP plane was 0.23 (0.4) cm deeper than the left one (*p* < 0.001). After CD depth of the right and the left TAP plane did not differ significantly. The body weight before pregnancy significantly affected the TAP distance (*p* <0.001, Fig. 3) and was the strongest independent factor, stronger than BMI or weight gain. Parity directly correlated with body weight, but was no independent factor for TAP distance in our model.

**Table 2:** distances in cm from the skin to the abdominal muscle layers

	Before CD		After CD	
	left	right	Left	right
EOM	1.8 (1.0)	2.0 (1.0)	2.1 (0.9)*	2.4 (1.1)*
IOM	2.5 (1.1)	2.6 (1.2)	2.9 (1.1)*	3.2 (1.3)*
TAM	2.9 (1.2)	3.0 (1.3)	3.7 (1.3)*	3.9 (1.4)*

All data presented as median (interquartile range). * = *p* < 0.001 before vs. after CD. *EOM* external oblique muscle, *IOM*internal oblique muscle, *TAM* transversus abdominis muscle, *CD* cesarean delivery

## Discussion

No systematic description of the TAP ultrasound anatomy in pregnant women exists in the literature. We studied the ultrasound anatomy of relevant structures in pregnant women before and after elective cesarean section. In this study, we found that the relevant muscular layers were visible in all ultrasound examinations before as well as after CD. The TAP was found to more superficial before CD and also the external and internal oblique muscles were located closer to the surface.

This finding may be explained by the specific changes in the abdominal wall during pregnancy. At term, due to the significantly increased volume of the uterus, the circumference of the abdominal wall is considerably increased. Accordingly, the layers of the abdominal wall are under tension and both subcutaneous fat as well as the muscular layers are thinned out. As a consequence, the TAP is shifted closer to the skin. Also, the firmness of the gravid uterus gives a good support for the ultrasound probe. With the surgical opening of the uterine cavity and delivery during cesarean section, the abdominal volume is decreased and the tension on the abdominal wall is released. Thus the slackened muscles and subcutaneous layer increase again in thickness.

A TAP block is a has been used in a variety of abdominal surgical procedures, including cesarean delivery [12, 16, 17]. If morphine is not used as intrathecal adjunct, like e.g. in Germany [18], a TAP block may be of potential benefit in this setting.

A TAP block for any surgical procedures may be initiated before or after the surgical procedure. Given the relatively slow onset of long acting local anesthetics, performing TAP block before surgery may potentially beneficial. However, in all randomized controlled studies on TAP block for postoperative pain control after cesarean delivery, the block was initiated after the surgical procedure [12, 19]. As shown in our study, the relevant muscular layers and the TAP compartment were visualized after delivery in all participants. Regarding distance of the TAP compartment to the skin administering the TAP block before delivery might be a meaningful strategy, as the target plane can be reached by the needle in a shorter distance than after CD, however, as local anesthetic systemic toxicity is a major concern with TAP blocks, we do not recommend such strategy. TAP associated seizure has been reported within 10 min of injection, thus it might have occurred before delivery, inflicting additional risk as compared to after delivery [20].

If the anesthetist decides to perform the TAP block after delivery it may be useful to perform an ultrasound examination of the abdominal wall preoperatively to minimize the time needed to establish the block after surgery, e.g. by preparing the ultrasound machines settings for focus depth and frequency. When employing this strategy one needs to keep in mind that, as seen in our study, the TAP shifts position after cesarean delivery. It is then found approximately 0.7 cm deeper in reference to the skin. Patient positioning also affects TAP depth, possibly because with left lateral displacement, the gravid uterus provides a firmer base for the ultrasound probe. Of note, we use 110 mm cornerstone needles for TAP blocks. With an angle of 45°, these would have been long enough for all studied patients.

The depth of the TAP depends on the body weight of the parturient. In patients with an increased BMI it can be expected to be observed deeper than average. The patient’s body weight should be taken into account when planning a postoperative TAP block. The depth and the frequency of the transducer may need to be adjusted and more time may need to be allocated for the procedure [21, 22]. Additionally, the choice of needle length may need to be optimized in this way too.

For transverse abdominal incisions as in CD, TAP block needs to be performed on both sides to obtain a bilateral effect. Bilateral TAP injections of ropivacaine have been shown to achieve relevant plasma concentrations of local anesthetic 30 min after injection [23]. Elevated plasma ropivacaine levels and symptoms of mild neurotoxicity have been observed after TAP block for cesarean sections [24]. Individual dosing strategies should be applied to minimize the risk of potentially toxic plasma concentrations [25]. Given the risk of relevant transfer of local anesthetic into the circulation, the sonographical anatomy of the TAP region should be judiciously visualized before puncture, while advancing the needle and during injection, in order to minimize the total applied dose of local anesthetic by poorly targeted injection. Also, patients must be monitored closely for at least 45 min after injection, as peak plasma concentration is reached about 30 min after injection [24]. The main limitation of this observational sonoanatomical study is that no actual TAP blocks were performed.

## Conclusion

Relevant anatomical landmarks for a TAP block are sonographically well visible in 100% of cases after cesarean delivery. After CD, median depth of the TAP was 3.8 (1.3) cm in the studies population. Depth increases by 0.04 cm per kg bodyweight before pregnancy. After CD, median depth of the TAP increases by 0.65 (0.1–2.2) cm, which might suggest to perform TAP Block before delivery. For safety reasons we do not suggest that strategy. Scanning the abdominal wall before CD will underestimate the target depth of the TAP after delivery. The obstetric anesthetist needs to be aware of these changes when planning a TAP block in the context of cesarean delivery.

## Abbreviations

BMI: Body mass index
CD: Cesarean delivery
TAP: Transversus abdominis plane
